# A Canadian Survey of Research on HIV-1 Latency—Where Are We Now and Where Are We Heading?

**DOI:** 10.3390/v16020229

**Published:** 2024-02-01

**Authors:** Ana Luiza Abdalla, Gabriel Guajardo-Contreras, Andrew J. Mouland

**Affiliations:** 1 HIV-1 RNA Trafficking Laboratory, Lady Davis Institute at the Jewish General Hospital, Montreal, QC H3T 1E2, Canada; ana.santos@mail.mcgill.ca (A.L.A.); gabriel.guajardo@mail.mcgill.ca (G.G.-C.); 2 Department of Microbiology and Immunology, McGill University, Montreal, QC H3A 2B4, Canada; 3 Department of Medicine, McGill University, Montreal, QC H4A 3J1, Canada

**Keywords:** HIV-1, AIDS, PLWH, cART, LRA, viral reservoirs, viral latency

## Abstract

Worldwide, almost 40 million people are currently living with HIV-1. The implementation of cART inhibits HIV-1 replication and reduces viremia but fails to eliminate HIV-1 from latently infected cells. These cells are considered viral reservoirs from which HIV-1 rebounds if cART is interrupted. Several efforts have been made to identify these cells and their niches. There has been little success in diminishing the pool of latently infected cells, underscoring the urgency to continue efforts to fully understand how HIV-1 establishes and maintains a latent state. Reactivating HIV-1 expression in these cells using latency-reversing agents (LRAs) has been successful, but only in vitro. This review aims to provide a broad view of HIV-1 latency, highlighting Canadian contributions toward these aims. We will summarize the research efforts conducted in Canadian labs to understand the establishment of latently infected cells and how this informs curative strategies, by reviewing how HIV latency is established, which cells are latently infected, what methodologies have been developed to characterize them, how new compounds are discovered and evaluated as potential LRAs, and what clinical trials aim to reverse latency in people living with HIV (PLWH).

## 1. Introduction

In Canada, the human immunodeficiency virus type—type 1 (HIV-1)—is far from eradicated. According to the last report from the Public Health Agency of Canada in 2022, it was estimated that around 68,000 people are living with HIV-1, with a national rate of 4.7 new infections per 100,000 inhabitants [1]. Geographically, these numbers are more alarming in certain provinces, being the highest in Saskatchewan and Manitoba, with rates of 19 and 13.9 per 100,000 inhabitants, respectively. In Canada, the prevalence of HIV-1 is higher in men—the number of cases is double compared with women—and the main routes of transmission are male-to-male sexual contact and drug injection [2].

When combinatorial antiretroviral therapy (cART) was first introduced, researchers believed that two years of treatment would be enough to eradicate HIV-1. Although cART efficiently suppresses viral replication, upon cessation, HIV-1 rebounds rapidly due to the presence of latently infected cells that are mostly transcriptionally inactive during cART. Long periods of treatment have shown that latently infected cells, herein called reservoirs, are extremely stable and have a half-life of approximately 44 months in patients under cART. Therefore, estimates suggest that it would take approximately 70 years of treatment to eliminate the HIV-1 reservoir, representing the biggest challenge in achieving a cure [3].

The HIV-1 reservoir is established rapidly following HIV-1 exposure [4]. To date, there are no approaches that efficiently target the latent provirus. Several aspects of HIV-1 latency need to be accounted for to implement any sterilizing approach, including transcriptional regulation of the HIV-1 DNA, the type of cells that harbor integrated provirus, and the tissue/niche where cells are located [5,6]. In this review, we discuss the state of the art on HIV-1 latency, focusing on the specific contributions from Canadian laboratories and their potential impacts on cure research.

## 2. HIV-1 Latency: The State of the Art 

Latency establishment involves the integration of the full-length HIV-1 proviral DNA into the host’s chromatin. Competent latency is not the case for all cells, as several do not harbor HIV-1 DNA that codes for fully infectious viruses. The proviral DNA can be defective due to 5′-, 3′-, or internal deletions, resulting in the production of noninfectious viral particles [7,8,9]. Although several cells could harbor integrated HIV-1, the competent latent reservoir is rare [10]. The mechanism by which infected cells establish a latent state is still open to debate, but evidence suggests that this is established early post-infection [11]. Aside, the location of the provirus’ integration into the host genome is also important as most of the latently infected cells have integrated provirus in transcriptionally silent regions, repressing the expression of the virus [12,13].

HIV-1 can infect and establish latency in several cell types, such as macrophages, microglia [14], follicular dendritic cells [15,16], and several subsets of CD4+ T cells [6]. Among the subsets of CD4+ T cells, central memory (T_CM_) and effector memory (T_EM_) are reported to be the main reservoirs harboring integrated HIV-1 and intact proviruses [6,17]. For this reason, it is believed that the virus infects CD4+ T cells in a short timeframe, where a small pool of activated T cells differentiates into long-term memory T cells when they are still susceptible to infection [18]. A key question in the field is where latency is established. Although latent cells can be present in the peripheral blood, they are in low proportions [19], suggesting that this is not the main HIV-1 reservoir contributing to the rebound of viremia following cART cessation [20]. Several sites have been identified as favorable reservoirs, such as the central nervous system (CNS) [21,22], gut-associated lymphoid tissues (GALT) [23], and lymph nodes [24,25]. Importantly, cells from privileged sites, such as the CNS, have been shown to receive suboptimal concentrations of cART and often harbor a defective proviral DNA, which results in the production and secretion of viral proteins that contribute to the chronic activation of the immune system [7,10,26].

## 3. Host and Viral Factors in Latency Establishment

The ways in which HIV-1 establishes latency remain topics of investigation and intense scientific discussion. The consensus is that the establishment of the HIV-1 latent reservoir occurs early during infection [5]. For example, a patient who initiated cART ten days post-HIV-1 exposure still experienced viral rebound following cART cessation, even after two years of treatment and having an undetectable viral load [11]. Studies in nonhuman primates (NHP) showed that latently infected cells were established in less than 72 h [27,28]. Additional evidence comes from post-exposure prophylaxis (PEP). The increased failure of PEP directly correlates with the time taken before starting cART [29,30,31]. Most government health agencies suggest taking PEP drugs within 72 h after potential exposure, and the reason comes from the evidence that, in the context of mucosal exposure, the virus takes up to 72 h to reach the lymph nodes [32].

Other factors that account for a productive infection are the cells’ inner function and activation status. For example, naïve CD4+ T cells are resistant to HIV-1 infection, while activated CD4+ T cells are more susceptible [33,34]. As part of the inherent immune response, T cells become activated upon infection. When activated cells die, only long-lived memory CD4+ T cells remain, representing the main HIV-1 latent reservoir (see Donahue et al., 2013 [35], and Dufour et al., 2020 [5] for details). Therefore, a key question is “at what point is latency established in CD4+ T cells?”. T cells may become infected upon cell activation, achieving latency when these cells turn into a memory phenotype. Another possibility could be that activated cells become infected during the transitioning from activated to the long-lived, quiescent memory phenotype [5]. Evidence from in vitro studies has shown that targeting specific molecules associated with certain cellular phenotypes could affect the number of cells that harbor latent HIV-1. Evans et al. showed that blocking the programmed cell death protein 1 (PD-1), an immune checkpoint associated with T cell activation/exhaustion, before HIV-1 infection decreased the number of latently infected cells [36]. Fromentin et al. complemented this observation as they observed that HIV-1 reactivation was enhanced when blocking PD-1. The engagement of PD-1 with its ligand (PD-L1) inhibits viral expression and impairs the T cell receptor (TCR)-induced HIV-1 reactivation in latently infected cells. Moreover, blocking PD-1 signaling with the monoclonal antibody pembrolizumab enhanced HIV-1 production in combination with bryostatin, without increasing T cell activation [37]. Additionally, they found that PD-1 expression is higher in latently infected CD4+ T cells in patients undergoing cART [37], suggesting that the PD-1 pathway could be involved in establishing and maintaining HIV-1 latency. However, other factors, aside from CD4+ T cell activation status, influence the susceptibility of cells. Casuso et al. showed that HIV-1 preferentially infects highly dividing CD4+ T cells, independent of their activation status [38]. Moreover, blocking specific metabolic pathways, such as glycolysis, dampens HIV-1 infection, leading to cell death, and could further affect the size of the latent reservoir [38].

Another aspect of the T cell profile that influences the establishment of latency is the subtype of CD4+ T helper (Th) cells [6]. Cleret-buhot et al. showed that the proportion of latency is higher in Th_17_ cells compared with Th_1_ cells. They identified MAPK3K4 and the tyrosine-protein phosphatase non-receptor type 13 (PTPN13) in Th_17_ cells as possible factors in latency establishment, as the downregulation of these proteins decreased HIV-1 DNA integration [39]. In addition, Kulpa et al. demonstrated that, after latency-reversing agents (LRA) stimulation, T_CM_ differentiated into T_EM_, the latter identified as one of the main HIV-1 reservoirs [40]. By ex vivo and in vitro approaches, Kulpa et al. also showed the association between the response to LRAs and the transcriptional pathways related to T cell differentiation, the acquisition of effector function, and the cell cycle [40]. In the natural course of an infection, T_CM_ cells are reactivated upon antigen stimulation, acquiring a T_EM_ phenotype [41]; however, it is not clear how that would happen in the context of latent HIV-1 infection.

Aside from host factors, viral factors also play a role in the latency establishment. Omondi et al. demonstrated a correlation between the HIV-1 protein Nef subtypes and the HIV-1 immune evasion and reservoir size. They showed that some Nef subtypes are more efficient than others in driving HIV-1 escape from the immune system, which positively correlates with the reservoir size, underscoring the fact that viral proteins could also influence the establishment of latency [42].

## 4. Molecular Mechanism of HIV-1 Latency

**Figure 1:**
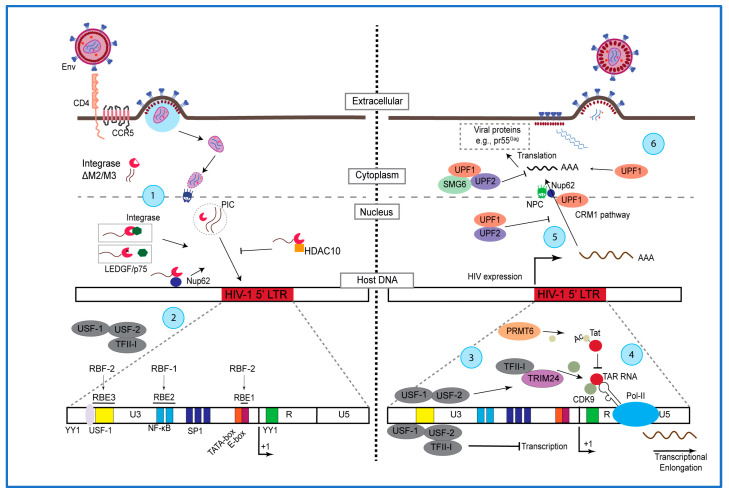
**Molecular mechanisms contributing to HIV-1 latency.** Early events of HIV-1 replication begin with viral Env binding to CD4 on the target cell, implication of co-receptors (e.g., CCR5), and fusion to the plasma membrane, and then release of the viral capsid into the cytoplasm. The translocation of the pre-integration complex (PIC) and integration of the viral DNA into the host DNA are shown. (1): Events related to the roles of host factors modulating integrase function. SUMO-interacting motifs, M2 and M3, leading to a blockade of PIC nuclear translocation. The binding of integrase to LEDGF/p75 or to a mutant (marked with an “×”) as well as to Nup62 and to HDAC10 are illustrated, and the effects on integration are indicated (an arrow or an arrow ending with a bar, indicating positive and negative regulation, respectively). (2): Cis-acting elements are shown within the HIV-1 LTR, including the RBE and RBF elements. (3): Association of host factor binding to the LTR and effects on HIV-1 transcription, highlighting the binding of TFII-I to TRIM24 and the recruitment of CDK9 and TAR RNA/Tat to RNA polymerase II (Pol-II) and acetylation (Ac) of Tat by PRMT6 (4). Indicated are the roles of UPF1 complexes in nucleocytoplasmic export of the viral RNA through the nuclear pore complex (NPC) (5) and their roles in the regulation of viral mRNA stability and viral protein expression (6). Please see the text for details.

HIV-1 latency is complex, and what occurs at a cellular level is not any different. From virus binding to host receptors (e.g., CD4), to the regulation of proviral DNA transcription, every step of the virus replication can be modulated to influence the state of HIV-1 latency (Figure 1). In addition to the classical role of the HIV-1 envelope protein in viral entry, the gp120 portion also has different roles. For example, when recognizing CD4 in an infected cell, HIV-1 gene expression is downregulated, presumably by modulating the HIV-1 long terminal repeat (LTR) [43]. In contrast, gp120 can also bind to the α4β7 receptor, promoting CD4+ T cell activation and proliferation, resulting in HIV-1 expression [44]. In addition to CD4, other receptors have been implicated in the regulation of the HIV-1 LTR activity. For example, CD45 counteracts the phorbol 12-myristate 13-acetate (PMA)-induced HIV-1 reactivation [45] and the engagement of CD43 [46] or exposure to virions expressing B7.2 induced the activation of HIV-1 LTR [47]. These examples illustrate how HIV-1 proteins modulate latency due to the low levels of virus expression in reservoirs.

Following HIV-1 fusion to the plasma membrane, the capsid is released into the cytoplasm. The viral RNA is reverse transcribed to double-stranded DNA and, along with the integrase and host proteins, forms the pre-integration complex (PIC), allowing the integration of the proviral DNA into the host genome [48]. The process of HIV-1 translocation to the nucleus involves the nuclear pore complex, which works as a barrier to regulate nucleocytoplasmic transport under physiological conditions [49]. Several viruses, including HIV-1, usurp this complex to promote nuclear translocation [49]. Most events preceding integration are related to the restriction of HIV-1 translocation. However, pre-integration latency may also occur in which the PIC remains intact but only upon reactivation is the viral DNA integrated [50] (Figure 1). Therefore, when considering HIV-1 latency, it is also important to consider the possibility of pre-integration events that contribute to the reservoir.

Along with the PIC, several host proteins assist in its interaction with the host chromatin. Perhaps the most studied is lens epithelium-derived growth factor (or LEDGF/p75), which binds to the viral integrase, licensing the PIC toward transcriptionally active sites [51,52,53]. Moreover, the viral integrase can be regulated by post-translational modifications, such as acetylation, that increase the integrase activity [54,55]. Another important modification is the SUMOylation, which consists of the addition of SUMO (small ubiquitin-like modifier) groups to lysine residues in target proteins. SUMOylation is important for the integrase function early in the replication cycle and operates independently of the binding to LEDGF/p75. Zheng et al. described how SUMOylated proteins can interact with each other through specific SUMO-interacting motifs (SIM), including the HIV-1 integrase [56]. The HIV-1 integrase has three SIMs (M1-M3). Mutations in the M2 and M3 motifs impaired nuclear translocation, as well as reduced binding to LEDGF/p75 [56]. Interestingly, the binding to Ku70, a nuclear protein involved in DNA repair, was enhanced in the M2 and M3 SIM mutants. The infection with a virus containing SIM mutations exhibited s nuclear translocation of the PIC [56]. Although this phenotype can not be precisely associated with LEDGF/p75 or Ku70, it is clear that SUMOylation of the HIV-1 integrase plays an important role in the PIC nuclear translocation (Figure 1).

Of note, previous work from Zheng et al. showed that LEDGF/p75 is dispensable for interaction between the HIV-1 integrase and the host chromatin. They observed that by mutating the C-terminal domain of the integrase, which binds to LEDGF/p75, the integrase maintained the ability to bind to the host chromatin and further integrate the viral DNA. However, two introduced mutations (EH170,171AA) in integrase failed to bind LEDGF/p75 but induced low levels of viral replication, suggesting that LEDGF/p75 has another important function in HIV-1 replication [57]. Although the result from this study seems contradictory to what has been previously shown, it does not necessarily invalidate previous findings as the authors did not evaluate the efficiency of integration or the integration site—thus, it remains unclear whether the mutations, specifically EH170,171AA, reduced overall integration of vDNA or led the PIC to transcriptionally inactive sites.

During nuclear translocation of the PIC, the nucleoporins Nup153, Nup98, transportin-3, and RanBP2/Nup358 play key roles [49]. Moreover, nucleoporins have additional roles once the PIC enters the nucleus. Ao et al. showed that Nup62 interacts with the HIV-1 integrase and chromatin, but that its depletion does not affect nuclear translocation of integrase. However, this reduced the binding of the integrase to chromatin and the levels of integrated vDNA, suggesting that Nup62 plays a role in the integration of viral DNA [58]. Other host factors that interact with the HIV-1 integrase and influence its activity are the histone deacetylases (HDAC), playing important roles in the establishment of HIV-1 latency. Ran et al. identified HDAC10 as a host factor that reduces viral gene expression, which is downregulated during the course of the infection. In addition, HDAC10 interacts with the integrase, and its depletion increased the levels of integrated viral DNA, independent of the integrase acetylation status [59] (Figure 1).

Latency reversal involves the expression of the integrated HIV-1 genome, which is driven by a single promoter located at the 5′ LTR. In short, the HIV-1 LTR is located between the nucleosomes nuc-0 and nuc-1. Sequentially, from 3′ to 5′, it comprises a canonical TATA box with an enhancer box (E-box), as well as binding sites for the transcription factors SP-1, NF-κB, and USF-1. Further details on the binding sites, cis and trans elements are extensively reviewed in Delannoy et al. [60].

Despite the cis elements being related to the canonical transcription factors driving the HIV-1 LTR (e.g., NF-κB, NFAT), other elements have been identified as important in regulating HIV-1 expression. Ras-responsive region binding factor-1 (RBF-1) and -2 (RBF-2) are factors required for HIV-1 expression as they bind to Ras-responsive binding elements (RBEs) found in the HIV-1 proviral DNA [61]. Initially, RBF-1 was found to bind to the ets-like motifs within the Ras-responsive binding element 2 (RBE2). RBF-2 was characterized as a complex containing USF-1/USF-2 and the general transcription factor II-I (TFII-I), which cooperatively binds to RBE1 within an E-box element adjacent to the TATA box and to RBE3 upstream the NF-κB binding sites [61,62,63]. By itself, USF-1 is associated with transcriptional activation [64], and when found in the RBF-2 complex, it suppressed transcription due to the recruitment of HDAC3 [65]. In parallel, Malcolm et al. highlighted the implications of RBF-2 in the regulation of the HIV-1 LTR activity, by demonstrating that both, TCR- and PMA-, but not TNF-α-induced HIV-1 expression through RBF-2 and the Ras/MAPK signaling [65,66]. A single T-A substitution at the 3′ of RBE3 weakened the binding of TFII-I, which impaired PMA-induced virus expression and the binding of RBF-2 to the HIV-1 LTR [65]. Although these findings appear contradictory, there is likely a balance between activation and repression based on structural changes and binding factors on the LTR (For example, RBF-2), although the exact mechanism remains unclear [65,66] (Figure 1). Further investigations in RBF-2 found that the tripartite motif protein 24 (TRIM24) acted as a cofactor of RBF-2 in the induction of transcriptional elongation of the HIV-1 provirus [67]. Horvath et al. found through siRNA assays that TRIM24 bound to TFII-I and assisted in inducing HIV-1 LTR activity. Moreover, they found that TRIM24 was important for recruiting the cyclin-dependent kinase 9 (CDK9) to promote elongation. In parallel, the depletion of TRIM24 favored the establishment of latency upon HIV-1 infection in Jurkat T cells [67]. Last, the transcription factor Yin Yang 1 (YY1) is a factor previously shown to bind downstream to the TATA box (−16 to +27) and to repress HIV-1 transcription [68]. Later, YY1 was shown to bind RBE3 in unstimulated conditions, while in PMA-induced conditions, YY1 dissociated from the HIV-1 LTR. Furthermore, YY1 recruited HDAC1 and promoted viral silencing during HIV-1 infection [69].

Among the other transcription factors that are important for HIV-1 expression, the CCAAT/enhancer-binding proteins (C/EBP) have three binding sites on the HIV-1 LTR [70]. Dumais et al. showed that prostaglandin-E2 increased cyclic adenosine monophosphate (cAMP) levels, which leads to the formation of a heterodimeric complex of C/EBP-β and the cAMP-response element-binding protein (CREB). This complex interacts with the proximal binding site in the HIV-1 LTR, to induce virus expression [71]. A recent study from Canchi et al. showed that the overall expression of C/EBP-β is increased in the brain of patients with HIV-1-associated neurocognitive disorders (HAND), in postmortem frontal cortex tissue. They demonstrated that C/EBP-β expression is modulated by the viral protein Tat and that C/EBP-β levels are reduced in neurons and increased in astrocytes compared with samples from PLWH with no cognitive disorders [72]. They associated their observations with the role of C/EBP-β in regulating inflammation, metabolism, and autophagy in astrocytes, suggesting that C/EBP-β plays a role in the development of HAND due to neuroinflammation.

The importance of NF-κB and the nuclear factor of activated T cells (NFAT) in inducing HIV-1 transcription is widely characterized. The regulation of the HIV-1 LTR is the endpoint that results from a cascade of protein activation. Despite the classical stimulus that leads to NF-κB- and NFAT-induced HIV-1 expression through protein kinase C (PKC) activation, or TCR engagement, respectively, there are other less studied pathways. Ryckman et al. found that the expression of S100A8, S100A9, and S100A12, known as myeloid-related proteins (MRPs), induced viral expression by activating NF-κB, eliciting a response through the enhancer region of HIV-1 LTR in J1.1 cells, a human T cell line latently infected with HIV-1 [73]. Additionally, Dahal et al. showed the relevance of the SR RNA-binding proteins. They demonstrated that the knockdown of the SR-RBP CLK1 increased HIV-1 promoter activity and enhanced the response to LRAs by increasing HIV-1 reactivation, while depletion of CLK2 suppressed it. In addition, by inhibiting CLK1 and CLK2, but not CLK3, they observed a suppression of HIV-1 gene expression [74], suggesting that CLK proteins regulate latency at different steps.

Importantly, the HIV-1 genome codes the transactivation of transcription (Tat), a critical regulatory protein responsible for binding to the trans-activation response element (TAR) in the nascent viral RNA to drive transcription elongation [75]. Several reports demonstrate that the regulation of Tat is essential to drive efficient HIV-1 expression [75,76]. Xie et al. showed that Tat methylation, mediated by the protein arginine N-methyltransferase 6 (PRMT6), affects Tat’s ability to recruit CDK9—a master regulator of RNA transcription—to impair HIV-1 transcriptional elongation [76]. Importantly, Tat is a regulatory protein that prevents the establishment of latency [77], as exogenous Tat, from another source/virus, can reactivate HIV-1 latently infected cells [35,77]. Despite that, Tat binding to TAR is essential, and additional evidence shows that other components in the HIV-1 promoter also contribute to successful Tat-induced virus expression. For example, Wilhelm et al. identified the GTGC sequence, flanking the TATA box in HIV-1 LTR, as essential for the recruitment of transcriptional factors to the PIC [78].

The full-length HIV-1 RNA is processed co-transcriptionally by the host splicing machinery to generate multiple RNA species that will be translated to produce Gag, Gag-Pol, Envelope, accessory, and regulatory proteins. Wong et al. showed that cardiotonic steroids (CS) play a role in HIV-1 RNA splicing, restricting HIV-1 expression through its interaction with the Na^+^/K^+^ ATPse [79]. They observed that CS led to an over-spliced HIV-1 RNA and nuclear retention, thereby reducing Gag synthesis from the full-length viral RNA. In addition to mRNA splicing, another way to regulate mRNA translation is via the activity of microRNAs (miRNA) [79]. Ouellet et al. demonstrated that the TAR RNA can be used as a template to generate microRNAs, identifying two miRNAs that regulate HIV-1 gene expression [80]. On the other hand, Pardons et al. determined that the host microRNA miR125b contributes to the maintenance of latency in resting CD4+ T cells, while this role was attributed to miR155 in effector memory CD4+ T cells, highlighting that latency regulation may vary between CD4+ T cell subtypes [80,81].

Our group has reported on the control of viral RNA processing and trafficking [82,83,84,85,86,87,88] and their possible impact on HIV-1 latency [85]. We identified that HIV-1 hijacks the Up frameshift 1 (UPF1) protein to increase the stability of the viral genomic RNA [82,85]. UPF1 has been implicated in RNA surveillance as it targets mRNAs for degradation through the nonsense-mediated mRNA decay (NMD) pathway, by forming a complex with other host cofactors, such as UPF2, UPF3a, SMG1, and SMG6 [89]. UPF1-mediated viral RNA stability is dependent on the binding to UPF2, suggesting that the role of UPF1 is independent of its primary role in the NMD pathway [82] (Figure 1). In addition, the overexpression of UPF1 increased the reactivation of the HIV-1 provirus in a latent T-cell model system. Furthermore, in the same model, overexpression of either SMG6 or UPF2 downregulated HIV-1 reactivation [85]. In both cases, binding to UPF1 was required. Noteworthy, the co-overexpression of UPF1 and UPF2 is sufficient to rescue HIV-1 expression, suggesting that UPF1 fine-tunes viral genomic RNA stability, contributing directly to the maintenance of HIV-1 latency. The nucleoporin Nup62, present in the nuclear pore complex (NPC), is involved in the integration of the viral DNA [58] and participates in the nucleocytoplasmic export of the HIV-1 viral mRNA [90]. Ajamian et al. identified that UPF1 associates with Nup62 to mediate viral RNA nucleocytoplasmic export [83]. UPF1-mediated viral RNA nuclear export depends on Exportin-1 (also known as CRM1), which is consistent with the observation that UPF1 co-immunoprecipitates with Rev, CRM1, DDX3, and Nup62 in the context of HIV-1 infection [83]. Moreover, while UPF1 is required to bind UPF2 to degrade mRNA through NMD, in the context of HIV-1, the binding of UPF2 to UPF1 impairs viral RNA nuclear export [91]. As mentioned above, UPF1 regulation of the viral RNA affects the ability of infected cells to be reactivated [85], so the regulation of viral RNA nuclear export harbors similar considerations in controlling HIV-1 expression and could also play a role in the maintenance of latency.

## 5. Targeting of HIV-1 Latent Reservoirs

A definitive cure against HIV-1 will require targeting all latently infected cells, with the final aim of stopping cART without the concern of viral rebound. The HIV-1 reservoir is present at a low frequency (0.000001% of the T cell population), but it is represented by a long-lived pool of cells, with an estimated half-life of 44 months [3]. In Canada, several studies were carried out to decipher which are the HIV-1 reservoirs. These studies concur that the reservoir is highly diverse, represented by different cell subtypes residing in several tissues. Initial research suggested that the HIV-1 reservoir was present only in resting memory CD4+ T cells. Current research demonstrates that several T cell subsets harbor an integrated provirus, contributing to the size of the HIV-1 reservoir. An extensive review of how different CD4+ T cell subsets contribute to HIV-1 persistence can be found in Fromentin et al. [6]. In their other work to identify cellular markers that are expressed by latently infected CD4+ T cells from patients under cART, Fromentin et al. demonstrated that cells that harbor proviral DNA have a higher frequency of the immune checkpoint markers, PD-1, TIGIT, and LAG-3, in combination or alone [92]. Although these markers were not specific to HIV-1 infected cells, further research to identify a specific cell marker may represent a valuable tool to target the HIV-1 reservoir.

Chomont et al. identified that T_CM_ and transitional memory T cell (T_TM_) CD4+ T cells represent major HIV-1 reservoirs, suggesting that cell self-renewal and proliferation play a role in viral persistence [17]. Interestingly, they observed that these two T cell subtypes defined two different reservoirs. T_CM_ are the main reservoir in patients with suppressed viral load and their CD4+ T cell counts are normal. T_CM_ do not markedly proliferate and are long-lasting. In contrast, the HIV-1 reservoir is found in T_TM_ in viremic patients with low CD4+ T cell counts, which are cells in constant proliferation due to persistent activation of the immune system [17]. Gantner et al. complemented the observations by demonstrating that the persistent HIV-1 reservoir in T_CM_ is due to clonal expansion, suggesting that the reservoir observed in other subtypes is derived from the progeny of T_CM_ after antigen-driven expansion [93]. Of note, both studies suggest that early adhesion to cART impacts the HIV-1 reservoir size, which goes hand in hand with more recent work from Massanella et al. This group recruited participants and grouped them based on the time of cART initiation after infection—less than 30 days, between 31 to 90 days, or greater than 90 days—and determined the pool of latently infected cells before and up to four years after cART initiation [94]. They observed that the HIV-1 reservoir in people who initiated cART the earliest was smaller compared with the other groups, thus suggesting that early detection and adhesion to cART plays a role in the size of the reservoir [94]. Importantly, different T cell subtypes also respond differently to treatments. Sannier et al. evaluated the effect of different LRAs in reactivating the integrated provirus in different T cell subtypes [95]. All the evaluated memory T cell subtypes efficiently reversed latency, but marked differences were observed depending on the specific LRA and the subtype of memory T cells. They observed that the T_EM_ subtype re-established HIV-1 protein synthesis, while the other memory subtypes showed a latency reversal mainly at the level of transcription, supporting that the HIV-1 reservoir is influenced by cell-intrinsic factors [95].

The HIV-1 reservoir is present in multiple tissues, encompassing the lymph nodes, GALT, CNS, spleen, lungs, liver, and bone marrow [20,96,97]. If cART is interrupted, HIV-1 can be reactivated from all of these sites, turning anatomical features into a key point to consider in future treatments (reviewed in detail by Costiniuk et al. [98]). The GALT harbors abundantly infected CD4+ T cells and macrophages [99]. Importantly, a significant T cell depletion after HIV-1 infection occurs in the GALT, which recovers slowly, even after restoring the CD4+ T cell levels due to strict adherence to cART [100]. The GALT is proposed as a “hideout” for HIV-1, as cART penetration is lower when compared with circulating cells [101]. Another site described as an HIV-1 reservoir is the lungs. Costiniuk et al. evaluated the presence of HIV-1 CD4+ T cells from pulmonary mucosa, obtained from bronchoalveolar lavage from infected adults under suppressive cART [102]. They found that proviral DNA is 13-fold higher in T cells from lung mucosa compared with the matched blood samples. They also observed that T_EM_ were the most abundant T cell subtype in the lungs from PLWH and concluded that the pulmonary mucosa represents an important tissue in establishing the HIV-1 reservoir, even under cART [102]. HIV-1 is also detected in the CNS as early as a week after primary infection, triggering an immune response and neuroinflammation [103,104]. Research suggests that HIV-1 crosses the blood–brain barrier by traveling within infected T cells and macrophages, but there is not enough evidence to strongly support this hypothesis, as most of it comes from postmortem samples. The CNS is still understudied, mainly due to the difficulty of accessing human samples that are not postmortem, with many conclusions being drawn from studies in animal models or in vitro cell culture [105]. Importantly, HIV-1 requires adaptation to effectively establish a reservoir in the CNS as brain-derived HIV-1 isolates are less dependent on CD4 levels at the cell membranes for entry, which has been proposed to be related to macrophage infection (microglia), which are more abundant than T cells in the CNS [106,107]. In addition to microglia, astrocytes of the CNS are latently infected by HIV-1. Barat et al. demonstrated that astrocytes are relevant to HIV-1 infection as proliferative astrocytes are readily infected when compared with non-proliferative astrocytes [108]. When they evaluated provirus reactivation in astrocytes, none of the evaluated LRAs were able to reverse latency, suggesting that astrocytes might not constitute an HIV-1 reservoir but produce low levels of viral proteins, which could contribute to the neurological disorders seen in PLWH [108]. In conclusion, HIV-1 persists and establishes a reservoir in different anatomical sites within the human body, posing a challenge to eradicating HIV-1, as each tissue has different kinetics for drug uptake and unique cellular factors that support or impede viral persistence.

## 6. Determination of the HIV-1 Reservoir Size

There are several methodologies to evaluate the percentage of latently infected cells or the percentage of HIV-1 reactivated cells obtained from blood samples from PLWH. Initial approaches were PCR-based assays, in which the integrated provirus was detected using specific primers against the HIV-1 LTR regions [109,110,111,112]. Unfortunately, even when these approaches provide quick and easy-to-interpret answers, they do overestimate the size of the reservoir due to the detection of defective proviruses [109,113]. To partially overcome this issue, the quantification of only replication-competent viruses was carried out by the quantitative outgrowth assay (QVOA) [114,115] or the Tat/Rev-induced limiting dilution assay (TILDA) [116]. The first methodology consists of CD4+ T cells isolation from PLWH and further reactivation of HIV-1, followed by co-culture with noninfected CD4+ T cells to allow HIV-1 spread. The readout is infectious units per million (IUPM) of CD4+ T cells, determined by the quantification of viral release by p24 ELISA or RT-qPCR [115,117]. The second methodology is based on the fact that the viral RNAs that code for the viral proteins Tat and Rev are present at low levels in latently infected cells. Their expression is high in HIV-replicating cells, using the presence of these mRNAs as a readout for viral reactivation [118,119,120]. To do this, CD4+ T cells are isolated from PLWH and then treated to induce HIV-1 reactivation. Later, samples are subjected to qRT-PCR to determine the abundance of *tat* and *rev* RNAs [116]. Although both techniques provide an idea of the reservoir size, there are limitations to consider. QVOA is an expensive and time-consuming technique, requiring between 10 and 15 days and large amounts of blood (~150 mL) to be carried out. It also relies on a single round of reactivation with a specific LRA, which does not reactivate all proviruses, underestimating the reservoir size [121]. TILDA is a faster technique, requiring only two days and 10 mL of blood, making it a useful technique in clinical research. However, TILDA still overestimates the reservoir size as it detects defective proviruses that still code for *tat* and *rev* viral RNAs [113,121]. In addition, none of the mentioned techniques can distinguish between different subsets of CD4+ T cells as they are all based on bulk-isolated cells.

More recently, a flow cytometry-based approach, with several contributions from Canadian researchers, has been developed to evaluate the HIV-1 reservoir size and, also, to determine which subset of cells are more prone to be latently infected [122,123,124,125]. Ivan Sadowski’s team attempted to study HIV-1 latency by flow cytometry using a double-labeled HIV-1-coding vector termed Red-Green HIV-1 (RGH). RGH allows the detection of HIV-1 by GFP expression as well as integration into the host genome by mCherry expression under the control of the constitutively expressed cytomegalovirus promoter. This allows the distinction between latently (GFP^−^/mCherry^+^) and productively (GFP^+^/mCherry^+^) HIV-1 infected cells [122]. Dahabieh et al. infected Jurkat T cells with RGH to understand whether latency is established early after infection or during later timepoints. They observed latently infected cells as early as two days post-infection, with no apparent increase in their proportion seven days after. They also confirmed that the RGH vector can infect activated primary CD4+ T cells from healthy donors, resulting in approximately 1% of cells latently infected on day six post-infection [122]. Finally, they showed that the latency established by the RGH vector can be reversed with commonly used LRA, supporting the double-labeled vector as a useful tool to study latency in vitro using flow cytometry [122]. Importantly, they observed a significant population of primary CD4+ T cells as GFP^+^/mCherry^−^, suggesting viral expression with no vector integration into the host genome. The authors attribute this to the silencing of the cytomegalovirus promoter due to T cell activation [122,126,127], proposing a future version with a constitutive promoter derived from a host gene instead of a viral sequence.

One of the main problems of HIV-1 detection by flow cytometry is that anti-HIV-1 antibodies have a high signal-to-noise ratio, preventing a reliable detection of HIV-1^+^ cells. This situation is overcome by FISH-Flow, a technique that integrates flow cytometry with fluorescence in situ hybridization (FISH), allowing the detection of RNA at a single-cell level [128,129]. Daniel Kaufmann’s group validated this technique for HIV-1 using samples from PLWH and a set of probes targeting the viral *gag* and *pol* mRNAs, along with an antibody against the HIV-1 Gag protein [125]. By FISH-Flow, the sensitivity of HIV-1 detection was improved significantly, detecting one thousand HIV-1^+^ cells per 10^6^ cells, compared with one HIV-1^+^ cell per 10^6^ when using single detection of viral mRNA or Gag [125]. In this work, Baxter et al. also characterized the subset of CD4+ T cells where HIV-1 was predominant. They determined that memory CD4+ T cells (central CD45RA^−^/CD27^+^, and effector CD45RA^−^/CD27^−^), isolated from the blood of PLWH during viremia, were the most frequently HIV-1 infected cells. In addition, these cells presented increased levels of exhaustion markers CTLA-4, PD-1, and TIGIT [125], consistent with previous publications [112,113,114,115]. Finally, they evaluated differences in CD4+ T cells between viremic and aviremic patients. They observed that memory CD4+ T cells carried most of the HIV-1 mRNA^+^/Gag^+^ cells in both groups, with viremic and aviremic patients having higher T_CM_ and T_EM_ proportions, respectively. In addition, they observed that the ability to reactivate HIV-1 with different LRAs shifts between the subtypes of memory T cells, even if the LRAs belong to the same class. For example, HIV-1 was reactivated from T_EM_ cells by bryostatin, but not by ingenol, while T_CM_ exhibited poor HIV-1 reactivation by bryostatin [125]. However, even when the FISH-Flow methodology proved to be a reliable and improved technique over previous methods, one of its major drawbacks is the requirement of a large number of cells (at least 1 × 10^7^ CD4+ T cells per patient). A detailed protocol for the HIV-1 FISH-Flow is publicly available [124].

Nicolas Chomont’s group developed a simplified version of flow cytometry by using a combination of anti-HIV-1 antibodies instead of mRNA probes, as detecting HIV-1 by at least two different antibodies should reduce the number of false positive events [123]. In this work, Pardons et al. targeted two different epitopes of the HIV-1 capsid protein, using the antibodies KC57 and 28B7. They isolated and activated CD4+ T cells from the blood of PLWH under viremic or aviremic conditions. As expected, they observed an increase in double-positive events in viremic patients compared with aviremic patients, which increased in both groups when treated with PMA/ionomycin [123]. Then, they determined which subset of CD4+ T cells was preferentially infected with HIV-1. They observed that effector memory CD4+ T cells (CD45RA^−^) were the main T cell subset infected with HIV-1, supporting previous observations [125]. Then, they compared CD4+ T cell phenotypes between viremic and aviremic patients. In aviremic patients, double-positive HIV-1 cells were present in the effector (CD45RA^−^/CCR7^−^/CD27^−^) and transition (CD45RA^−^/CCR7^−^/CD27^+^) memory CD4+ T cells. In viremic patients, HIV-1 was found in T_CM_ (CD45RA^−^/CCR7^+^/CD27^+^) and Th17 cells (CCR4^+^/CXCR3^−^/CCR6^+^) [123], the latter reported as highly permissive to HIV-1 infection [115,116]. The authors also observed that HIV-1 infected cells also expressed high levels of the α4β1 integrin receptor in the blood samples from both viremic and aviremic individuals. This integrin drives the migration of cells toward the inflamed CNS and to the bone marrow, supporting their identity as an HIV-1 reservoir [123]. The flow cytometry approach to measure the HIV-1 reservoir has proven to be as sensitive as the QVOA or TILDA assays, detecting one double-positive event every 10^6^ CD4+ T cells, with an improved R^2^ when combining the two antibodies to detect HIV-1. Importantly, this approach reduces by half the number of cells required per patient and requires less time to be carried out in comparison to the FISH-Flow approach, underscoring the usefulness of this tool to phenotype and understand the HIV-1 reservoir [123].

## 7. Therapeutic Strategies

Strategies to target HIV-1 from latently infected cells have been evaluated thoroughly by research teams in Canada (Table 1). In 2015, Zhu et al. determined the efficiency of targeting different portions of the HIV-1 provirus by CRISPR/Cas9 [130]. This system utilizes short nucleotide sequences (termed guide RNAs) to recognize the complementary DNA and cleave it, generating double-stranded DNA breaks that, after repair, cause insertions or deletions at the target site [131,132]. Zhu et al. evaluated the inactivation of the integrated HIV-1 provirus in J-Lat 10.6 cells, a T-cell line model used to study HIV-1 latency [133]. They designed ten different guide RNAs, targeting conserved sequences present at the HIV-1 LTR, the polymerase (pol), or the regulator of expression of viral proteins (rev) segments within the HIV-1 genome. Following transfection, they stimulated HIV-1 expression by adding TNF-α and then measuring HIV-1 reactivation by quantifying viral release into the supernatant [130]. All guide RNAs reduced the amount of viral release, with a guide RNA targeting the second exon of Rev as the most effective, proving in vitro the feasibility of targeting and inactivating the HIV-1 reservoir by CRISPR/Cas9 [130].

Dr. Jonathan Angel and his group developed a strategy to target HIV-1 latently infected cells based on their previous research. Initially, they showed that latently infected cells exhibited an impaired IFN response [134], followed by the fact that the oncolytic virus, Maraba virus (MG1), targets IFN-I-defective tumors in vivo with minimal toxicity [135,136]. Considering this, they evaluated the use of MG1 to target HIV-1 latently infected cells by infecting the promonocytic cell lines U937 and U1 with different multiplicity of infections (MOI) of MG1 (U1 being a U937-derivative cell line where the HIV-1 provirus has been integrated) [137]. They observed that using an MOI of 0.005, 80% of U1 cells were infected with MG1, while only 20% of the U937 cells became infected. Consequently, cell survival was 60% in MG1-infected U1 cells, while no significant cell death was observed in infected U937 cells [137]. Based on this, they evaluated the feasibility of the MG1 strategy in CD4+ T cells isolated from healthy donors, followed by HIV-1 infection. Three days after infection, they proceeded to infect the cells with MG1 and to determine the size of the HIV-1 reservoir. In agreement with their previous results, MG1 infection decreased the amount of detected HIV-1 provirus and viral release from infected CD4+ T cells after latency reversal with phytohemagglutinin (PHA) and IL-2, suggesting that MG1 could be a feasible therapy to target HIV-1 latently infected cells. However, they required an MG1 MOI of 10 to observe significant differences [137].

More recently, Mann et al. evaluated a polyvalent virus-like particle formulation, designated activator vector (ACT-VEC), as an anti-HIV-1 vaccine that will reactivate the latent HIV-1 provirus [138]. The formulated vaccine was developed by cloning the full-length sequence of the HIV-1 provirus from the serum of five PLWH before cART initiation [139]. The five genomes were cloned into vectors and engineered to produce non-replicative HIV-1 virus particles. Later, monocytes were isolated from a group of nine PLWH, under cART since the early stages of infection, to further pulse monocyte-derived dendritic cells with the ACT-VEC formulation. Pulsed dendritic cells were exposed to purified autologous CD4+ T cells ex vivo to evaluate levels of HIV-1 reactivation [138]. An ACT-VEC pulse induced a significantly higher reactivation compared with PMA/ionomycin treatment, a common combination used to reverse latency in vitro [140]. Importantly, they observed that the released ACT-VEC-derived viruses did not show a significant genetic diversity, supporting the use of the ACT-VEC formulation as a vaccine candidate that will target CD4+ T cells expressing HIV-specific TCRs [138].

In addition to the targeting strategies of the latent HIV-1 reservoir, the development of latency-modifying agents is an important therapeutic research axis. These agents comprise LRA, the “shock and kill” approach [141], and latency-promoting agents (LPAs), commonly associated with the “Block and Lock” approach [142], both associated with two HIV-1 curative strategies. There are significant and ongoing efforts in Canadian laboratories to screen and find LRA or LPA compounds. A spotlight of these investigations is the compounds derived from natural sources, like marine sponges, other invertebrates, and microorganisms [143,144]. Thirteen LRAs and one LPA were identified in various screenings. In addition, eight were found to reactivate HIV-1 in screening the libraries: kinase inhibitor library [145], DIVERSet [146], and pANAPL (pan-African natural product library) [147]. All these compounds acted through different mechanisms to modulate viral expression (Table 1).

**Table 1:** Overview of screenings for latency modifying agents.

First Author (Year)	Compounds	Mechanism	Methodology/Comments	Source from Screening	Ref
**Latency-Reversal Agents (LRA)**					
Wang, 2016	Sesterpenoid alotaketal C, D, and E. Ansellone A	PKC activation	Screened in J-lat 9.2 A total of 9 compounds extracted	Marine sponge- *Phorbas* sp.	[143]
Wang, 2022	Ansellone J Phorone C	PKC activation	Screened in J-lat 9.2 A total of 5 compounds extracted	Marine sponge- *Phorbas* sp.	[144]
Ao, 2016	PKC412	Activation of NF-κB signaling pathway	Screened in ACH2 cells	Small molecules library (ChemBridge) and kinase inhibitor library (BML-2832-0100)	[145]
Hashemi, 2016	PH01, PH02, PH03, PH04, and PH05	N.E. * iκB degradation (PH02 only)	Jurkat cells infected with HIV-luciferase reporter virus	180.000 small molecules from 3 libraries (CCBN, LCGC and DIVERSet)	[146]
Richard, 2018	Psammaplin A, Apllysiatoxin, Debromoaplysiatoxin	HDACi Or PKC Activation	J-lat 8.4 and 10.6; PBMCs from PLWH	Marine invertebrates and microorganisms. Library from R.J.A lab ^$^	[148]
Richard, 2020	Knipholone anthrone Anthralin	Tat deacetylation or influence on the PKA pathway	J-lat 9.2 and PBMCs from PLWH	pANAPL A total of 216 screened compounds	[147]
Fortin, 2000	bis-peroxo- vanadium (bpV) PTP inhibitors	Constant NFAT activation	Jurkat cells and PBMCs from health donors	NA *	[149]
Hovarth, 2023	IACS-9571	Increase binding of TRIM24 to HIV-1 LTR	Jurkat cells expressing mHIV-luc and Hek293T	NA *	[150]
Bernhard, 2011	Chaetocin	Reduced H2K9 methylation	Jurkat cells infected with HIV-luciferase reporter virus	NA *	[151]
Planas, 2020	T007090	Inhibition of PPARy	Isolated CD4+T cells differentiated into Th17 in vitro and CD4+ T memory cells from PLWH	NA *	[152]
**Latency-Promoting Agents (LPA)**					
Tietjen, 2018	Bengamide A	NF-κB signaling	CEM CD4+T cells	A total of 252 screened compounds from marine invertebrates	[153]
Schonhoger, 2021	Flavopiridol	CDK9 inhibitors		Flavanoids	[154]
**Other Approaches**					
Zhu, 2015	CRISPR/Cas9	HIV-1 silencing with gRNAs for pol and rev	J-lat 10.6 reactivated with TNF-α and quantified viral release in the supernatant	NA *	[130]
Ranganath, 2018	MG1—Maraba Virus	Killing of latent infected cells	Latent infected derived U937 macrophages and primary CD4+T cells infected in vitro	NA *	[137]
Mann, 2020	ACT-VEC—polyvalent virus B-clade derived	Reactivation of latent HIV	Ex vivo exposure of primary latent infected CD4+T cells from PLWH to ACT-VEC	NA *	[138]

* NA: Not Applicable; NE: Not evaluated; ^$^ RJA lab: Richard James Anderson lab.

## 8. Clinical Trails

According to Health Canada, there are 74 clinical trials related to HIV-1 (website: https://health-products.canada.ca/ctdb-bdec/; accessed on 13 January 2023). Of them, only four evaluate a treatment to reduce the reservoir size in PLWH. In 2006, Routy et al. carried out a clinical trial [155,156] based on the evidence that chromatin remodeling plays a role in HIV-1 latency [157]. They performed an open-label, randomized clinical trial to evaluate the effect of Valproic acid (VPA) in combination with cART on the HIV-1 reservoir size as VPA is a histone deacetylase inhibitor. They determined the reactivation of the HIV-1 provirus after 16 or 48 weeks of daily administration of 1000 mg of VPA plus cART. The study included 42 PLWH, all with suppressed viral load for at least a year and a CD4+ T cell count of at least 200 cells/mm^3^. They observed a decline in the number of latently infected cells after 16 weeks of VPA+cART treatment, but there were no statistical differences compared with control conditions (cART-only) or with 48 weeks of co-treatment [156] (NCT00289952).

The recombinant growth hormone has been a treatment to improve immune function and CD4+ T cell reconstitution in PLWH under cART [158]. In 2017, a proof-of-concept study evaluated the effect of the recombinant human growth hormone (rhGH) on the size of the HIV-1 reservoir (NCT03091374). They assessed if the combinatory treatment of cART and rhGH could improve PLWH CD4+ T cell function and reduce HIV-1 reservoir size. They co-treated 22 PLWH with 3 mg/day of rhGH on top of cART for 24 weeks, followed by half the dose of rhGH for 24 additional weeks. All patients achieved viral suppression for at least two years and a CD4+ T cell count of at least 350 cells/mm^3^. Up to date, no results regarding the treatment efficacy have been released.

PLWH under prolonged cART can develop metabolic complications that contribute to persistent immune activation related to a low CD4+/CD8^+^ T cell ratio [159]. T cell immunometabolism regulates cell proliferation [160]; therefore, improving this could enhance T cell function and control the size of the HIV-1 reservoir [161,162]. Trautmann et al. showed that HIV-1-specific CD8^+^ T cells have an altered metabolic state related to immune activation and exhaustion [163]. In 2018, Routy et al. set up a clinical trial to evaluate metformin as an immunometabolic agent, combined with cART, to reduce the HIV-1 reservoir in PLWH [31]. Metformin, a well-tolerated drug commonly used to treat type II diabetes [164], inhibits mTOR signaling by activating the AMP-activated protein kinase (AMPK) pathway, promoting autophagy and improving CD4+ T cell counts in diabetic PLWH under cART [161,162].; They recruited 22 nondiabetic PLWH with suppressed viral load for at least three years and a CD4+/CD8^+^ T cell ratio below 0.7. They treated them with 500 mg of metformin plus cART for 12 weeks, followed by an additional 12 weeks of only cART. The study aimed to evaluate and compare the reservoir size and CD4+/CD8^+^ T cell ratio at the baseline, as well as 12 and 24 weeks after co-treatment initiation (NCT02659306). Unfortunately, metformin treatment did not decrease the amount of integrated HIV-1 DNA or the reservoir size measured by TILDA in CD4+ T cells from peripheral blood mononuclear cells (PBMCs), even though 8/13 study participants showed a reduction in the HIV-1 RNA/DNA ratios [165].

Last, a previous study reported that the simian immunodeficiency virus (SIV) preferentially infected CD4+ T cells that express the α4β7 integrin receptor on their surface. This receptor is a lymphocytic homing receptor that is involved in cell trafficking to the GALT, a site where HIV-1 leads to a severe depletion of CD4+ T cells during acute infection [166,167]. Byrareddy et al. observed that targeting CD4+ T cells with a monoclonal antibody against α4β7 (Vedolizumab) improved viral remission and decreased the viral load in the GALT of SIV-infected macaques during two years [166]. In 2020, McGuinty et al. developed a pilot study to assess the translation of this treatment with Vedolizumab in PLWH [168]. They enrolled 12 patients under suppressive cART for at least two years and a CD4+ T cell count of at least 500 cells/mm^3^. Subjects were divided into three groups and treated with a low, middle, or high dose of Vedolizumab (75 mg, 150 mg, and 300 mg, respectively), administered once a month for five months, followed by seven months of no treatment. Two months after the first administration, subjects would stop cART and proceed only with the administration of Vedolizumab (NCT03147859). Samples would be collected at the baseline and at the fifth and thirteenth month after treatment initiation to assess HIV-1 viral load and measure the reservoir size in GALT. No results from this clinical trial are available at the time of publication.

## 9. Concluding Remarks

HIV-1 latency is a complex process that involves viral and cellular factors, diverse T-cell subtypes, and several anatomical sites. Even though a better understanding has been achieved in the field of HIV-1 latency, many gaps remain to successfully target the latently integrated provirus. Here, we provide an extensive overview of contributions to this field of endeavor by Canadian teams of investigators (summarized in Table 2). We maintain an optimistic outlook, and the development of an HIV-1 cure should be an attainable goal in the future.

## Figures and Tables

**Table 2:** Canadian contributions to the understanding of HIV-1 latency.

First Author of Study	Year	Main Findings *	Reference
**Molecular Mechanisms in HIV-1 Transcriptional Regulation**			
Clemente-Estable, Mario	1996	HIV-1 5’ LTR variants show different binding to the transcription factors c-Ets1 and Elf1.	[169]
Clemente-Estable, Mario	1998	RBF-2 binds to HIV-1 5’ LTR and represses its transcription.	[61,62]
Barbeau, Benoit	2001	NFAT1-mediated HIV-1 proviral expression is downregulated by CD45.	[45]
Barat, Corinne	2002	CD43 ligation to the TCR induces T cell activation and HIV-1 proviral expression.	[46]
Malcolm, Tom	2007	HIV-1 proviral expression requires the RBF-2 and Ras/MAPK signalings.	[65,66,170]
Xie, Baode	2007	HIV-1 Tat methylation by PRMT6 decreases HIV-1 transcriptional activation.	[76]
Dahabieh, Matthew	2011	An E-box motif within the HIV-1 LTR promoter is the binding site for the RBF-2 complex (USF1, USF2, and TFII-I).	[63]
Wilhelm, Emmanuelle	2012	CTGC DNA motifs flanking the HIV-1 TATA box are required for the correct formation of HIV-1 PIC.	[78]
Bernhard, Wendy	2013	YY1 transcription factor binds to the HIV-1 LTR to promote viral latency.	[69]
Rao, Shringar	2018	The RNA surveillance protein UPF1 is a positive regulator of viral reactivation, while UPF2 and SMG6 are negative regulators.	[85,86]
Wong, Raymond	2018	Cardiac glycoside inhibits HIV-1 gene expression through modulation of MEK1/2—ERK1/2 signaling.	[79]
Planas, Delphine	2020	Inhibition of PPARy in Th17 cells activates HIV-1 transcriptional expression.	[152]
Dahal, Subdha	2022	Different SR kinases play distinct roles in HIV-1 provirus expression.	[74]
Horvath, Riley	2023	TRIM24 regulates HIV-1 proviral expression by interacting with the RBF-2 complex, mediating RNA Pol II phosphorylation and recruitment of CDK9.	[67]
Chomont, Nicolas	2009	Central memory and transitional memory CD4+ T cells are the major HIV-1 reservoirs in infected people.	[17]
**HIV-1 Reservoirs in the Human Body**			
Boulassel, Mohamed-Rachid	2012	Determining CD4+ T cell nadir significantly predicts levels of HIV-1 proviral DNA.	[171]
Fromentin, Rémi	2016	CD4+ T cells harboring integrated HIV DNA express the immune checkpoint molecules PD-1, TIGIT, and LAG-3.	[92]
Huang, Yiying	2016	Testis restricts the distribution of cART due to the differential expression of drug transporters.	[172]
Asahchop, Eugene	2017	cART drugs show variable concentrations and efficacies in brain myeloid cells and tissues in a drug-specific manner.	[173]
Barat, Corinne	2018	A small fraction of astrocytes are able to carry silent HIV-1 proviruses.	[108]
Costiniuk, Cecilia	2018	The pulmonary mucosa is highly enriched in HIV-1 latently infected CD4+ T cells.	[102]
Omondi, Frederick	2019	HIV-1 Nef subtype diversity influences the reservoir size, correlating subtype B to the largest viral reservoir.	[42]
Brumme, Zabrina	2019	Perinatally infected participants who experienced longer durations of uncontrolled viremia have increased HIV-1 reservoir size.	[174]
Proust, Alizé	2020	HIV-1-infected astrocytes accumulate a very high amount of Aβ, and the LRA bryostatin-1 induces a reduction in their endocytosis, while JQ1 treatment results in a very slow degradation of them.	[175]
Gantner, Pierre	2020	Single-cell TCR sequencing reveals that clonal expansions highly contribute to the persistence of the HIV reservoir.	[93]
Mohammadzadeh, Nazanin	2021	HIV-1 and SIV persist in brain tissues despite undetectable virus in the blood during cART.	[176]
Massanella, Marta	2021	Participants who initiate cART within 30 days or less of HIV-1 infection have a more sustained decay in the reservoir size.	[94]
Sannier, Gérémy	2021	Single-cell profiling reveals that LRAs efficiently induce transcription in all memory cell subsets but translation mostly in effector memory cells, rather than in the long-lived central memory pool.	[95]
Turcotte, Isabelle	2023	HIV-1 patients with coronary artery atherosclerotic plaques display larger reservoirs.	[177]
Dufour, Caroline	2023	Full-length HIV-1 sequencing revealed that multiple copies of identical HIV genomes are found in all tissues, indicating that clonal expansions are common in anatomical sites.	[178]
**Techniques to Study HIV-1 Reservoir Size**			
Dahabieh, Matthew	2013	Double-labeled HIV-1 vector that allows for detection of infected cells independently of LTR activity.	[122]
Hashemi, Farhad	2016	A mini-dual HIV reporter virus to study early latent or productive infections.	[179]
Salahuddin, Syim	2019	Methodology for processing bronchoalveolar lavage fluid and matched PBMCs.	[180]
Pardons, Mario	2019	Flow cytometry-based assay to quantify and characterize HIV-1 infected cells produced by combining two antibodies targeting the viral capsid.	[123]
Sannier, Gérémy	2021	Flow cytometry combined with in situ RNA hybridization to detect HIV-1 viral genes and the viral capsid.	[95]
Di, Yunyun	2022	How to induce HIV-1 latency in the TKO-BLT mouse model.	[181]
**Immune Response Modulation in the Context of HIV-1 Latency**			
Fortin, Jean-françois	2004	The HIV-1 proteins Tat and Nef mediate immune hyperactivation by increasing IL-2 expression.	[182]
Thibault, Sandra	2009	Toll-like Receptor 5 stimulation induces NF-ƘB, which reactivates latently infected T cells.	[183]
Dahabieh, Matthew	2014	NF-ƘB activity during the time of infection is an important regulator for HIV-1 nonproductive infection and viral integration.	[184]
Masroori, Nasser	2016	The IFN-I-induced gene PML transcriptionally silences HIV-1 in mouse embryonic fibroblasts.	[185]
Baxter, Amy	2016	The differentiation status of CD4+ T cells affects LRA effectiveness	[125]
Ranganath, Nischal	2016	HIV-1 latently infected macrophages and T cells show defects in IFN-I response.	[134]
Tietjen, Ian	2018	The natural product bengamide A inhibits NF-ƘB-dependent HIV-1 reactivation.	[153]
Jin, Steven	2019	Nef sequences from elite controllers displayed a lower ability to inhibit the TCR-mediated T cell activation by NFAT transcription factor.	[186]
Fromentin, Rémi	2019	Immune checkpoint blockers may facilitate latency disruption in infected individuals under cART.	[37]
**Latency-Reversing Agents (LRAs)**			
Bernier, Richard	1995	Parasite *Leishmania donovani* reactivates HIV-1 latently infected macrophages.	[187]
Dumais, Nancy	1998	Prostaglandin E2 secretion by macrophages can activate latently infected T cells.	[71,188]
Bounou, Salim	2001	Virions bearing the host protein CD80 and anti-CD3 antibody activate HIV-1 proviral expression.	[47]
Ryckman, Carle	2002	Myeloid-related proteins induce HIV-1 reactivation in infected T cells.	[73]
Chen, Adrienne	2003	*Neisseria gonorrhoeae* induces HIV-1 reactivation through an NF-ƘB-dependent mechanism.	[189]
Bernhard, Wendy	2011	The histone methyltransferase inhibitor SUV39H1 induces HIV-1 proviral expression.	[151]
Donahue, Daniel	2012	Tat overexpression on T cells reduces the number of latently infected cells.	[35,77]
Zhu, Weijun	2015	HIV-1 provirus can be inactivated by targeting the HIV-1 LTR, pol, or Rev segments with CRISPR/Cas9.	[130]
Baxter, Amy	2016	LRAs effectiveness differs between CD4+ T cells, depending on their differentiation status.	[125]
Ao, Zhujun	2016	The kinase inhibitor PKC412 stimulates viral transcription in latently infected cells.	[145]
Wang, Meng	2016	Screening from *Phorbas* sp. Marine sponge compounds with potential LRA activity.	[143]
Ranganath, Nischal	2017	Use of the oncolytic Virus MG1 to target latently infected cells by targeting IFN-I deficient cells.	[137]
Mujib, Shariq	2017	Combination of Nef blockade and CD8+ T cell expansion as a strategy to target HIV-1 latently infected cells.	[190]
Ran, Xiaozhuo	2017	Soluble HIV-1 envelope is able to stimulate viral transcription in latently infected T cells.	[191]
Dental, Clélia	2017	Prostratin and bryostatin-1 induce a proinflammatory response in human cerebral cells, affecting CD4+, CD8+ T cells, and monocyte adhesion and transmigration in vitro.	[192]
Hashemi, Pargol	2018	Five novel compounds identified bioinformatically were tested as LRAs. In particular, PH02 combined with PEP005/ingenol-3-angelate show promising latency-reversing activity.	[146]
Richard, Khumoekae	2018	Screening for novel LRAs from a library of marine natural products. Four induced expressions of latent HIV-1 provirus in both cell line and primary cell models.	[147,148]
Kulpa, Deanna	2019	The differentiation of T cells into an effector memory phenotype facilitates HIV-1 reactivation by LRAs.	[40]
Pardons, Mario	2019	Different subsets of memory T cells respond differently to LRAs.	[81]
Divsalar, Donya	2020	Screening and evaluation of novel histone deacetylase inhibitors as HIV-1 LRAs. Four identified to inhibit HDAC activity and/or reverse HIV latency in vitro.	[193]
Mann, Jamie	2020	Polyvalent virus-like particle formulation from different HIV-1 sequences as a strategy to reactivate HIV-1 latently infected T cells.	[138]
Roda, Weston	2021	Development of a mathematical model to predict the effect of LRAs in HIV-1 and SIV brain infection.	[194]
Schonhofer, Cole	2021	Flavonoid screening detects a CDK9 inhibitor that acts to block HIV-1 latency reversal.	[154]
Hany, Laurent	2022	Macrophages and T cells respond differently to LRAs, bryostatin-1, romidepsin, and JQ1.	[195,196]
Horvath, Riley	2023	The TRIM24 inhibitor IACS-9571 promotes productive HIV-1 expression and delays the formation of latently infected cells.	[150]
**Clinical Trials on HIV-1 Latency**			
Smith, Kimberly	2010	Treatment with recombinant growth hormone modestly improved the naive CD4+ T cell count in HIV-infected patients on cART.	[158]
Routy, Jean-Pierre	2012	Valproic acid in combination with cART to reduce the size of the HIV-1 reservoir in CD4+ T cells. No significant reduction.	[156]
Routy, Jean-Pierre	2019	Metformin in combination with cART to reduce the size of the HIV-1 reservoir. Ongoing trail. Unpublished results.	[31,197]
McGuinty, Michaeline	2020	Vedolizumab (anti-α4β7 integrin) in combination with cART to evaluate viral suppression beyond cART. Ongoing trail. Unpublished results.	[168]

* Not all findings are discussed in the main text and the reader is referred to the cited reference.

## Data Availability

Not applicable.

## Abbreviations

ACT-VEC	Activator vector
AIDS	Acquired immunodeficiency syndrome
AMP	Adenosine monophosphate
AMPK	AMP-activated protein kinase
C/EBP	CCAAT/enhancer-binding protein
cAMP	Cyclic adenosine monophosphate
cART	Combined antiretroviral therapy
CDK9	Cyclin-dependent kinase 9
CNS	Central nervous system
CREB	cAMP-response element binding protein
CRISPR	Clustered regularly interspaced short palindromic repeats
CRM1	Chromosomal maintenance 1
CS	Cardiotonic steroids
CTLA-4	Cytotoxic T-lymphocyte protein 4
DDX3	DEAD-box helicase 3
Env	Envelope
FISH	Fluorescent in situ hybridization
Gag	Group-specific antigen
GALT	Gut-associated lymphoid tissue
HAND	HIV-associated neurological disorders
HDAC	Histone deacetylases
HIV-1	Human immunodeficiency virus—type I
IFN	Interferons
IUPM	Infectious units per million
LAG	Lymphocyte-activation gene
LEDGF	Lens epithelium-derived growth factor
LPA	Latency-promoting agent
LRA	Latency-reversing agent
LTR	Long terminal repeat
MAPK	Mitogen-activated protein kinase
MAPK3K4	Mitogen-activated protein 3-kinase 4
MG1	Maraba virus strain MG1
MOI	Multiplicity of infection
MRPs	Myeloid-related proteins
NFAT	Nuclear factor of activated T-cells
NF-κB	Nuclear factor kappa B
NHP	Nonhuman primates
NMD	Nonsense-mediated mRNA decay
NPC	Nuclear pore complex
pANAPL	pan-African Natural Product Library
PBMCs	Peripheral blood mononuclear cells
PD-1	Programmed cell death protein 1
PD-L1	Programmed cell death-ligand 1
PEP	Post-exposure prophylaxis
PHA	Phytohemagglutinin
PIC	Pre-integration complex
PKC	Protein kinase C
PLWH	People living with HIV
PMA	Phorbol 12-myristate 13-acetate
PRMT6	Protein arginine N-methyltransferase 6
PTPN13	Tyrosine-protein phosphatase non-receptor type 13
QVOA	Quantitative outgrowth viral assay
RBE	Ras-responsive binding element
RBF	Ras-responsive binding factor
RGH	Red-green HIV-1 vector
rhGH	Recombinant human growth hormone
SIM	SUMO-interacting motif
SIV	Simian immunodeficiency virus
SMG	Serine/threonine-protein kinase
SR-RBP	Serine and arginine-rich RNA-binding protein
SUMO	Small ubiquitin-like modified
TAR	Trans-activation RNA response element
T_CM_	Central memory T cell
T_EM_	Effector memory T cell
T_TM_	Transitional memory T cell
TCR	T cell receptor
TFII-I	General transcription factor II-I
TIGIT	T cell immunoreceptor with Ig and ITIM domains
TILDA	Tat/rev induced limiting dilution assay
TNF-α	Tumor necrosis factor alpha
TRIM24	Tripartite motif-containing 24 protein
UPF1	Up frameshift protein 1
USF-1	Upstream stimulatory factor 1
VPA	Valproic acid
YY1	Transcription factor Yin Yang 1
